# Chapter Oral Health Advocates: A Nationwide Model for Pediatrician Peer Education and Advocacy about Oral Health

**DOI:** 10.1155/2013/498906

**Published:** 2013-10-21

**Authors:** Charlotte W. Lewis, Lauren Barone, Rocio B. Quinonez, Suzanne Boulter, Wendy E. Mouradian

**Affiliations:** ^1^Division of General Pediatrics, University of Washington and Craniofacial Center, Seattle Children's Hospital, Seattle, WA 98195, USA; ^2^Division of Pediatric Practice, American Academy of Pediatrics, Elk Grove Village, IL 60007, USA; ^3^Schools of Medicine and Dentistry, University of North Carolina, Chapel Hill, NC 27599, USA; ^4^Department of Pediatrics, Dartmouth Medical School and New Hampshire Dartmouth Family Medicine Residency Program, Concord, NH 03301, USA; ^5^Schools of Dentistry, Medicine, and Public Health, University of Washington, Seattle, WA 98195, USA

## Abstract

*Objective*. (1) To describe an innovative program training US pediatricians to be Chapter Oral Health Advocates (COHAs). (2) To provide insight into COHAs' experiences disseminating oral health knowledge to fellow pediatricians. *Patients and Methods*. Interviews with 40 COHAs who responded to an email request, from a total of 64 (62% response). Transcripts were analyzed for common themes about COHA activities, facilitators, and barriers. *Results*. COHAs reported positive experiences at the AAP oral health training program. A subset of academic COHAs focused on legislative activity and another on resident education about oral health. Residents had an easier time adopting oral health activities while practicing pediatricians cited time constraints. COHAs provided insights into policy, barriers, and facilitators for incorporating oral health into practice. *Conclusions*. This report identifies factors influencing pediatricians' adoption of oral health care into practice. COHAs reported successes in training peers on integrating oral health into pediatric practice, identified opportunities and challenges to oral health implementation in primary care, and reported issues about the state of children's oral health in their communities. With ongoing support, the COHA program has a potential to improve access to preventive oral health services in the Medical Home and to increase referrals to a Dental Home.

## 1. Introduction

Dental caries is the most common chronic disease of childhood and significantly impacts children's well-being [1]. Among US children aged from 2 to 5 years of age, more than 25% have caries, a prevalence which appears to be on the rise [2]. Yet, young children, particularly those who are low-income, encounter substantial barriers accessing dental care for prevention or treatment of dental caries. Meanwhile, primary care physicians (PCP) who care for children in the US, specifically pediatricians and family physicians (and in some settings, nurse practitioners and physician assistants), have unique opportunities to deliver oral health anticipatory guidance and implement dental caries primary prevention at frequent well-child-care visits early in a child's life. Infants, young children, and their parents will likely see their PCP as many as 13 times before they have ever visited a dentist, and more children have ready access to primary medical care than to dental care, particularly if they are publicly insured [3].

Studies indicate that, with training, physicians can effectively deliver preventive oral health services [4, 5]. In an effort to encourage PCPs to further their involvement in oral health as a means to diminish oral health disparities among children, 44 out of 50 US states now reimburse PCPs to provide preventive oral health care services to Medicaid-enrolled children. However, until recently, most pediatricians have lacked formal training in oral health [6, 7] that would allow them to effectively deliver these services and bill for them.

Acknowledging the impact of dental disease on children's health and the unique role that pediatricians can play in addressing oral health beginning in infancy, the American Academy of Pediatrics (AAP) added oral health promotion to its strategic plan in 2006 and set about developing plans to educate US pediatricians about oral health using a train-the-trainer model. Funding for these efforts was provided by a grant from the American Dental Association Foundation. The result was the Chapter Oral Health Advocate (COHA) program, in which 1-2 representative pediatricians were recruited from each AAP chapter to become peer-to-peer educators—called COHAs—for fellow pediatricians in their state or AAP chapter (larger states have multiple chapters). COHAs were trained at the Chapter Advocacy Training on Oral Health (CATOOH), a 1.5-day course held 3 times (2008, 2009, and 2010) at AAP headquarters in Elk Grove, Illinois, USA. Following the CATOOH, COHAs implemented (or refined) an oral health preventive program within their own practices and then disseminated the model to their fellow pediatricians and other pediatric providers using strategies and techniques they had learned during the CATOOH and, subsequently, within their own practices.

This study describes participants' experiences during the CATOOH and subsequent implementation during activities as COHAs. We were specifically interested in roles that COHAs assumed and the opportunities and challenges that COHAs faced in their efforts to disseminate oral health knowledge and skills to other pediatricians. We intend that findings from this project will (1) allow refinement, expansion, and replication of the COHA program; (2) increase awareness of pediatric oral health issues that arise in primary care practice; (3) describe factors that influence pediatricians' willingness and abilities to adopt oral health into their routine and practice; (4) inform future models of physician peer training and advocacy that could be applied in other countries and to other areas within health care.

## 2. Methods

### 2.1. COHA Recruitment

The AAP recruited 20 volunteer pediatricians to attend the first CATOOH in 2008. Fifteen more COHAs were trained in 2009 and 36 in 2010. At the time of the interviews (during March 2011–February 2012), there were 64 COHAs from 50 states and US territories. The COHA program is ongoing and COHAs remain active in their roles and continue to expand their knowledge around oral health and increase their level of advocacy for children. COHAs are not paid and do not receive any funding from the AAP for their activities.

### 2.2. Training

A steering committee of pediatricians, dentists, and staff from the AAP and the American Dental Association (ADA) planned the CATOOH, which included didactic, interactive small group and hands-on sessions. Most of the faculty were pediatric dentists. Design of the CATOOH was based on principles of adult learning and evidence that a combination of didactic and interactive CME activities is substantially more effective than didactic sessions alone in promoting behavior change [8]. The 2008 CATOOH covered basic oral health science, fluoride, oral health risk assessment, prevention and anticipatory guidance, oral health reimbursement, and hands-on practice in oral examination and fluoride varnish application. In 2009 and 2010, the agenda was supplemented with presentations from previously trained COHAs about lessons learned and best practices and with a session on billing (see Table 1 for 2010 CATOOH agenda). In addition to attending the CATOOH, COHAs completed an online oral health training program, called *Protecting All Children's Teeth*, which was developed by pediatricians and dentists working together with the AAP (“PACT” is available at: http://www2.aap.org/ORALHEALTH/pact/index-cme.cfm). They also received directed readings, supply lists, resources for peer and patient education, and a list of state dental contacts.

At the end of each CATOOH, participants completed a commitment-to-change contract that specified at least 4 training sessions per year would be done by each COHA. Additionally, individual COHA goals included working with state Medicaid programs around PCP oral health reimbursement, educating residents and other trainees about oral health, link with oral health coalitions, and improving medical/dental relationships. After each CATOOH, the AAP offered technical assistance and organized an electronic listserv for COHAs to share ideas, strategies, and support and for research updates and announcements.

### 2.3. Qualitative Evaluation

The AAP and the University of Washington Institutional Review Boards (IRB) approved this project. A semistructured script was used with questions in the following categories: motivation to become a COHA, previous oral health experience, perceptions about the CATOOH, activities undertaken as a COHA, facilitators and barriers encountered by COHAs, and recommendations for the future.

All 64 COHAs were contacted by email and invited to participate in this study. After the initial email, 2 other emails were sent to nonrespondents. The first 8 interviews were conducted in person during a COHA Advisory Council meeting and the remainder by telephone. Consent to audiotape each interview was obtained. Interview duration ranged from 15 to 70 minutes. Each audiotape was reviewed, the content wase categorized into themes, and representative quotes was selected. To reduce bias, the interviewer (CWL) was not involved with planning or implementation of the CATOOH or the COHA program. Findings, including themes and representative quotes, were presented, and feedback about accuracy and completeness was elicited from the CATOOH Steering Committee and COHA Advisory Council.

## 3. Results

### 3.1. Subjects

Forty COHAs responded and were scheduled for an interview (62% responses after 3 emails). There were 9 men and 31 women. Participants had graduated from medical school an average of 17 years prior to the interview (range 4 to 44). Approximately one-quarter of subjects were in private practice, one-quarter practiced at a community health center or Federally Qualified Health Center, and one-half were academic pediatricians. Most COHAs practiced in suburban or urban locales while approximately 10% worked in rural settings.

### 3.2. Motivations to Become a COHA

Approximately two-thirds of interviewees had previous oral health involvement prior to becoming a COHA. These individuals volunteered to be COHAs because of their interest in oral health, which usually was motivated by their patients' oral health problems and difficulties accessing dental care. The other third of COHAs had no previous oral health experience, but most were involved with their local AAP chapter and/or other advocacy activities such as “Reach out and Read [9].” Some of those interviewed confessed an initial lack of interest in oral health prior to attending the CATOOH, as this participant stated, “I really wasn't that interested (in oral health) but when they asked for volunteers to be a COHA, no one volunteered so I figured, ‘Ok, I'll go. It's a trip to Chicago… .'” However, the COHA training proved influential and this same individual went on to say, “I came out of the 2008 CATOOH and was really excited about (oral health) and I was on fire about how we could do this with pediatricians.”

### 3.3. COHA Roles and Activities

Towards the goal of optimizing children's oral health, COHAs advocated pediatricians' role to be that of providing preventive oral health anticipatory guidance, screening for caries risk and dental disease, applying fluoride varnish to children at high risk for dental caries, and facilitating access to a Dental Home.

#### 3.3.1. Peer-to-Peer and Other Outreach Roles and Activities

Almost all of the interviewees met their goal of conducting at least 4 oral health training sessions per year and most did more. In general, COHAs felt the on-site training that they provided to other pediatricians and their staff was well received. As fellow pediatricians, COHAs were uniquely able to relate to those that they were training but COHAs also acknowledged that each pediatric practice is different, and thus, an individualized approach to training was necessary. For example, in some practices, the pediatrician applies the fluoride varnish whereas, in other practices, fluoride varnish application is delegated to another health care provider, such as the medical assistant. In addition to academic detailing and on-site training, COHAs used other ways to reach out to pediatricians in their state/chapter to provide education, usually by email or presenting grand rounds at their hospital or area medical schools. Some COHAs were able to make a greater impact by focusing time and energy at a state government level, for example, meeting with state Medicaid directors to advocate for PCPs' reimbursement for oral health services and for expanded access to dental care for poor and low-income children.

#### 3.3.2. Unique Academic COHA Roles and Activities

Academic COHAs, meaning those who work at universities and their affiliated medical centers and who typically have both clinical and educator roles, explained that their positions allowed them more time to spend on oral health activities than clinicians in private practice since it was expected that they would be involved in community projects, outreach, and trainee education. Most academic COHAs provide pediatric medical care for a disproportionate share of children with special health care needs, publicly insured and uninsured children. They also had regular contact with medical students and residents. There were common lessons that academic COHAs sought to impart to trainees: (1) oral health is part of well-child-care; (2) oral health prevention is easy to do; (3) it is important for pediatricians to partner with dentists in their community.

Additionally, academic COHAs gave resident noon lectures about oral health, developed a continuity clinic oral health curriculum, and incorporated an oral health module into the residents' community and/or advocacy rotation. These COHAs reported that residents had little difficulty incorporating oral health into their visits with patients. Interviewees attributed residents' ease with oral health to a few factors including that residents have additional time to spend with patients and that residents are still in the process of developing their routine. Referring to oral health, one COHA said, “Residents just do it if you tell them to.”

### 3.4. Facilitators to Successful COHA Activities

Although there was variation in the infrastructure in place to support COHAs, most COHAs listed 5 factors that enabled their success as COHAs: (1) the CATOOH; (2) support from the AAP, fellow COHAs, and others; (3) personal experience implementing oral health into their practice; (4) relationships with dentists; (5) reimbursement for oral health services.

#### 3.4.1. The CATOOH

Interviewees made overwhelmingly positive comments about their COHA training. In addition to knowledge gained at the CATOOH, COHAs learned from other COHAs' successes and failures, were given valuable resources like flip charts to use when educating fellow pediatricians, and developed strategies for developing collaborative relationships with dentists, expanding pediatrician involvement in oral health and optimizing billing for these services. Furthermore, the lectures and discussions with dentists at the CATOOH helped COHAs appreciate the expertise of their dental colleagues and made dentists in general seem “more approachable.” The most valued aspects of the CATOOH was the “hands-on” aspect of the training, meaning that COHAs were able to examine and apply fluoride varnish to actual children. COHAs found their experience at the CATOOH to be empowering as this COHA said, “All of a sudden it hit me. This is a doable, cost-effective thing.”

#### 3.4.2. Support from the AAP, Fellow COHAs, and Others

COHAs, whether they were new to oral health or previously involved, came away from the CATOOH highly motivated to promote oral health involvement among fellow pediatricians and to improve the oral health of children. It was important, COHAs expressed, to maintain this momentum upon return to the COHA's home states and to have a forum for “ongoing collaboration and exchange of ideas.” To that end, after each CATOOH, the AAP national office maintained regular contact with the COHAs. COHAs also worked with their local AAP chapter and stayed in touch with fellow COHAs via the listserv. Through these interactions, COHAs could avail themselves of expert assistance when problems or questions arose and were able to share resources and ideas. Most COHAs commented positively on the support they received from their local AAP chapter and its executive director who often helped with outreach and legislative contacts, as one COHA explained, “(The executive director) did a lot so that I could focus on outreach rather than organizing.”

Some COHAs worked in communities and states where there was a preexisting oral health coalition with whom they could work and rely on for additional support. In settings with limited resources, a few COHAs applied for small grants, usually from foundations, to offset the costs of some of their oral health activities. One COHA used Americorp volunteers, who helped in developing and maintaining detailed online and printed lists of local dentists' contact information, accepted insurance plans, and wait times for new appointments.

#### 3.4.3. Personal Experience Implementing Oral Health Services

After returning from the CATOOH, COHAs focused their initial efforts on incorporating oral health into their own practice and, in the process, learned a variety of lessons, as this comment reflects:

 Once you have done about 20 to 30, it becomes part of your routine. You are not clumsy anymore… (You need to) do it whether you are running behind or not. Otherwise you are only going to do it on days when it easy.

COHAs believed that their “insider” perspective provided them with ease and credibility in talking to fellow pediatricians and helped them be more positive about the process of integrating oral health into primary care as these quotes reveal:

 On paper it looks complicated. You need a pediatrician who has done it to make it doable.
 At first it takes you 3-4 minutes, but if you incorporate oral health into the history and the oral screening exam into your physical and then put the fluoride varnish on while you're examining the child's mouth, then you're done and it takes 60 seconds once you are used to it.

COHAs without prior oral health experience seemed to have a better sense about how the average pediatrician might be resistant to undergoing oral health training and adopting oral health into his/her practice. For example, one COHA remarked “If you don't know anything about (oral health), then you don't understand the magnitude of the problem and you don't know how easy it is, so you just think you can't add one more thing to your plate.” When COHAs had overcome such barriers personally, they felt they were more effective in encouraging other pediatricians to become involved.

#### 3.4.4. Relationships with Dentists

Some COHAs practiced in areas where dentists were already involved in training physicians about oral health, giving the opportunity for COHAs to participate and provide the pediatrician perspective. For example, one COHA who partnered with a dentist for such presentations noted:

 The dentist knows the science but he does not really know how a pediatric office works and what are going to be the barriers for pediatricians. When our state Medicaid program tried to roll this out without pediatrician involvement, none of the pediatricians was really sure they wanted to do it because, (after the dentist's presentation), the pediatricians did not know what would be involved, couldn't see how easy it was (because there was no hands-on demonstration), and that billing would be easy for the (pediatrician's) billing staff.

Additionally, most COHAs met with local dentists to discuss their role as a COHA and, in doing so, were also able to explicitly address fears that PCPs were “going to be practicing dentistry.” COHAs found that dentists were more supportive than expected once they found out that the pediatricians were focused on caries primary prevention in infants and young children (whom general dentists are often uncomfortable seeing, COHAs said). Meeting with dentists as a COHA allowed the pediatrician-dentist relationship to expand into a more collaborative one in which COHAs felt greater ease referring patients to and consulting dentists about specific cases. Positive experiences with dentists gave COHAs greater confidence in educating fellow pediatricians about the importance of children having a Dental Home. Even in settings in which access to regular dental care was limited, almost all of the COHAs had developed and shared strategies with fellow pediatricians for obtaining urgent dental care for their patients with acute dental problems. To that end, COHAs each knew a few dentists whom they could call upon for dental emergencies or more urgent treatment needs, as this COHA described:

 When I see rotten teeth, I call the dentist and make the appointment for the family. (When I ask them personally), they will never turn me down.

#### 3.4.5. PCP Oral Health Services Reimbursement

In most states, COHAs could rely on the fact that “the hook is that it is a procedure that pediatricians can do and get paid for.” The reimbursement was particularly attractive in states such as Washington and North Carolina, where Medicaid payment to PCPs for delivering oral health services ranges from $50 to $70 per encounter. However, the average payment is $15–$25 and most state Medicaid programs only reimburse for fluoride varnish application. Oral screening, risk assessment, and family education are expected but in most states not paid separately.

### 3.5. Barriers to COHA Activities

COHAs encountered 3 levels of barriers related to oral health dissemination to fellow pediatricians: (1)  personal professional barriers that interfered with achievement of goals they had set for themselves; (2) policy and colleague-level barriers, in the form of pediatrician reluctance to undergo training about oral health; (3) community-/patient-level barriers, which affected pediatricians' abilities to optimally address their patients' oral health needs.

#### 3.5.1. Personal Professional Barriers

The most often cited personal barrier faced by interviewees was lack of time to accomplish the activities they envisioned for themselves as COHAs. The economic climate may have worsened this situation for some COHAs who had acquired more clinical duties or lost administrative time as their respective institutions dealt with budget shortfalls. The majority of COHAs had little or no funding and few resources. When asked what would help them do more, COHAs typically replied, in one form or another, more time, more money, and more help.

#### 3.5.2. Policy and Colleague-Level Barriers

Fellow pediatricians would sometimes decline COHAs offer to conduct an office-based training, citing limited time and being overwhelmed with other demands. As one COHA put it, “They worry about the 1500 other things we have to do for *Bright Futures* (guidelines for health supervision).” There were other concerns among pediatricians, including some of the COHAs themselves, surrounding reimbursement for oral health activities and fluoride varnish application being only available for Medicaid-insured children. Although low-income children are considered at higher risk for dental disease, some COHAs stated that they wanted delivery of oral health services to be based on individual need, regardless of a child's insurance and were “uncomfortable doing it for one population and not another.” Logistically, it was often challenging to identify and direct services only to Medicaid-insured children in offices that served a mix of privately and publicly insured children (which is the norm for pediatricians in private practice in the US). There were also unique, state-specific barriers that made incorporating oral health into pediatricians' practices more challenging. For example, some states required that PCPs undergo oral health training in person with a dentist in order to qualify for Medicaid reimbursement. This requirement imposed burdens of time away from practice, need to travel, and lack of physician perspective.

#### 3.5.3. Community-/Patient-Levels Barriers

COHAs and their fellow pediatricians encountered barriers to educating families about oral health. In some communities, there was limited oral health literacy, which hindered families seeking regular professional dental care and practicing home oral hygiene. However, the main barrier to optimizing their patients' oral health was limited access to quality professional dental care, as this COHA described:

 Part of the (COHA) program is to encourage (pediatrician) referral to dentists and the standard question (from the pediatrician) is, “To whom do I refer?” And you do not always have an answer.

Barriers to dental care access for Medicaid-insured children were reported commonly. Most COHAs described that, within their communities, privately insured children typically went to pediatric dentists in private practice, but such care was unavailable to lower-income children because these dentists did not accept Medicaid. More often than not, low-income children received dental care at community health centers or increasingly, at for-profit dental clinic chains that were geared exclusively towards Medicaid-insured children. COHAs expressed concerns, which were based upon comments made by their patients' parents, about what seemed to be lower quality of dental care delivered at some of these chain clinics.

## 4. Discussion

This is the first paper to describe a national program of peer-to-peer physician education and advocacy about oral health. The previous literature reported that pediatricians perceive preventive oral health as within their purview [7] and that PCPs are capable of delivering preventive oral health services [10], described state's efforts at oral health integration into primary care [4, 11, 12], and demonstrated that PCP efforts result in improved oral health among their patients [10]. However, past efforts to train pediatricians about oral health have been limited to single states and have not always included pediatricians in the planning and delivery of educational programs. In this project, COHAs from every chapter were recruited for a national training program with the expectation that they would return to their home state and educate/train fellow pediatricians to deliver preventive oral health services, thus allowing for more widespread, standardized, and rapid dissemination. Furthermore, pediatricians were involved in developing and revising the CATOOH, and COHAs learned from one another's experiences. Utilizing pediatricians to train other pediatricians was considered essential because COHAs uniquely understood how pediatric practices function and how pediatricians could incorporate oral health into their routines.

COHAs provided at least 4, and sometimes substantially more, oral health training sessions per year to fellow pediatricians, as well as at grand rounds and local conferences. COHAs' original goals expanded and evolved once they returned to their home state and better understood the needs of their fellow pediatricians and children within their state. For some COHAs, this meant focusing more on their efforts on state-level advocacy or on promoting collaborative relationships with dentists. It was harder than anticipated to get practicing pediatricians to make time for training. Because of this, COHAs sometimes had to rely on strategies other than traditional academic detailing to reach fellow pediatricians (e.g., emailing pediatricians about web-based oral health training opportunities). Yet, COHAs then lost the advantage of a personal visit during which they could facilitate hands-on training. Resources that could allow COHAs to expand their efforts might include funding for administrative support and outreach efforts and assistance in developing alternative training strategies to accommodate PCPs' limited time.

These COHAs formed the eyes and ears for the status of children's oral health within their communities. They often encountered disparities in access to quality dental care that deserved further attention. Collectively, COHAs could bring attention to these issues, but they need to know how to quantify and direct their concerns. Furthermore, a number of COHAs had access to preexisting data, such as Medicaid claims and other reports, which, with additional technical assistance, would allow for tracking of oral health outcomes.

Pediatric residents' adoption of oral health was viewed as successful by the COHAs who supervised them. Ideally, having an established oral health routine when one finishes residency means that such routines will be sustained. Family Medicine is farther along in this process than any other medical specialty; their residents complete formal, standardized education in oral health as a required part of their training [13].

Finally, COHAs made important inroads in developing collaborative relationships with dentists in their communities, a process that was encouraged by positive interactions with dentists during the CATOOH. Such interdisciplinary relationships enhance professional learning, improve patient care, and ideally, promote improved access to dental care for children. When it was difficult for pediatricians' patients to access dental care, it was more challenging for pediatricians to fully implement preventive oral health into their practices. This was because pediatricians lacked any place to refer patients for ongoing dental care or when oral health problems were identified on screening examination. Partnering with programs such as Washington's ABCD (Access to Baby and Child Dentistry) [14], which trains dentists to care for young children and provides enhanced Medicaid reimbursement to do so, would satisfy the critical link of a professional dental care referral source that PCPs need to be truly effective in promoting oral health for all children.

It is important to acknowledge the limitations of this work. While employing qualitative methods allowed themes to emerge that otherwise might not have been considered in a traditional survey, the time-consuming nature of the interview may have discouraged some COHAs from responding. A response rate of 62% is reasonable for surveys of physicians but those who did not respond may have had different experiences or perspectives that are not reflected in this paper.

## 5. Conclusions

This paper describes a novel nationwide effort to train pediatricians to be oral health peer educators and advocates. It also provides insight into the varying roles COHAs played and the opportunities and challenges that COHAs and their fellow pediatricians encountered integrating oral health into well-child-care visits. Some barriers identified in these interviews are modifiable, such as by streamlining training requirements for PCPs to bill Medicaid for oral health services, whereas other barriers are more difficult to overcome, for example, time constraints among PCPs. Nevertheless, COHAs and their fellow pediatricians found that, once initiated, providing oral health services takes less time than anticipated and delivers a valuable service to their patients. Difficulty finding a Dental Home for patients who lack private dental insurance or cash to pay out-of-pocket is a known barrier to promoting preventive oral health within pediatric practice [11]. However, results from this study point to the potential for national- and local-level collaboration between dentists and physicians as a means to expand interdisciplinary education and collegiality, as well as to expand access to professional dental care for all children.

Though COHAs largely felt their efforts in reaching out to and training their peers had been successful, the increasing time pressure in pediatric practice creates a need for the most efficient training strategies; these could include use of social media, web-based resources, oral health prompts in the electronic medical record, and incentivizing training by pairing maintenance of certification with oral health quality improvement efforts. The relative ease with which pediatric residents adapted to implementing oral health services highlights the need for focused efforts in medical school and residency to ensure that new physicians receive sufficient didactic and clinical training in oral health.

This paper, focused on training and implementation, is the first in a series looking at the impact of the COHA program. Results provide insight into factors that bear consideration when asking physicians to incorporate preventive oral health care into medical practice. These include the importance of (1) pediatrician involvement in designing and delivering oral health educational programs; (2) beginning oral health education early in medical training; (3) individualizing the approach for each physician practice; (4) expanding oral health surveillance and advocacy capacities; (5) incorporating dental partnerships into every level of implementation. Applying the lessons learned in this study along with ongoing technical and financial support, the COHA program holds promise to further improve access to preventive oral health services in pediatric medical practices, diminish oral health disparities, inform oral health policy, coordinate state-level oral health surveillance and quality improvement initiatives, and enhance referrals and collaboration with dental professionals.

## Figures and Tables

**Table 1:** 2010 CATOOH agenda (November 5-6, 2010) and session type (didactic, interactive, or practical).

Introduction, target audience and learning objectives, and pretest
General sessions
(1) The tooth and nothing but the tooth: why are we here? (d)
(2) The importance of early oral health (d)
(3) Introduction to oral health risk assessment and fluoride varnish application (i)
(4) Hands-on workshop—oral screening examination, risk assessment, and fluoride varnish application (p)
(5) Fluoride modalities and their appropriate use (i)
(6) Oral health prevention and anticipatory guidance (i)
(7) Building collaborative relationships to ensure patients' access to a Dental Home (i)
(8) Strategies for reaching out to colleagues and changing behavior (i)
(9) Making the commitment and signing the “commitment-to-change” contract (i)
(10) Overcoming barriers: COHAs in action. (i)
(11) You can do it! Where do we go from here? (i)

Concurrent session
(1) Billing and payment for oral health services (i)
(2) Quality improvement, *Bright Futures*, and recertification (i)
(3) Triage and Dental Home options (i)

Posttest, evaluation

d: didactic format, i: interactive, and p: practical (hands-on).
